# Intrinsic and extrinsic drivers of shape variation in the albatross compound bill

**DOI:** 10.1098/rsos.230751

**Published:** 2023-08-16

**Authors:** Joshua Tyler, David P. Hocking, Jane L. Younger

**Affiliations:** ^1^ Milner Centre for Evolution, Department of Life Sciences, University of Bath, Claverton Down, Bath BA2 7AY, UK; ^2^ School of Biological Sciences, Monash University, Melbourne, Victoria, Australia; ^3^ Zoology, Tasmanian Museum and Art Gallery, Hobart, Tasmania, Australia; ^4^ Institute for Marine and Antarctic Studies, University of Tasmania, Battery Point, Tasmania 7004, Australia

**Keywords:** bill, albatross, allometry, diet, morphospace

## Abstract

Albatross are the largest seabirds on Earth and have a suite of adaptations for their pelagic lifestyle. Rather than having a bill made of a single piece of keratin, Procellariiformes have a compound rhamphotheca, made of several joined plates. Drivers of the shape of the albatross bill have not been explored. Here we use three-dimensional scans of 61 upper bills from 12 species of albatross to understand whether intrinsic (species assignment & size) or extrinsic (diet) factors predict bill shape. Diet is a significant predictor of bill shape with coarse dietary categories providing higher *R*^2^ values than dietary proportion data. We also find that of the intrinsic factors, species assignment accounts for ten times more of the variation than size (72% versus 6.8%) and that there is a common allometric vector of shape change between all species. When considering species averages in a phylogenetic framework, there are significant Blomberg's *K* results for both shape and size (*K* = 0.29 & 1.10) with the first axis of variation having a much higher *K* value (*K* = 1.9), reflecting the split in shape at the root of the tree. The influence of size on bill shape is limited, with species assignment and diet predicting far more of the variation. The results show that both intrinsic and extrinsic factors are needed to understand morphological evolution.

## 1. Introduction

**Figure 1.**
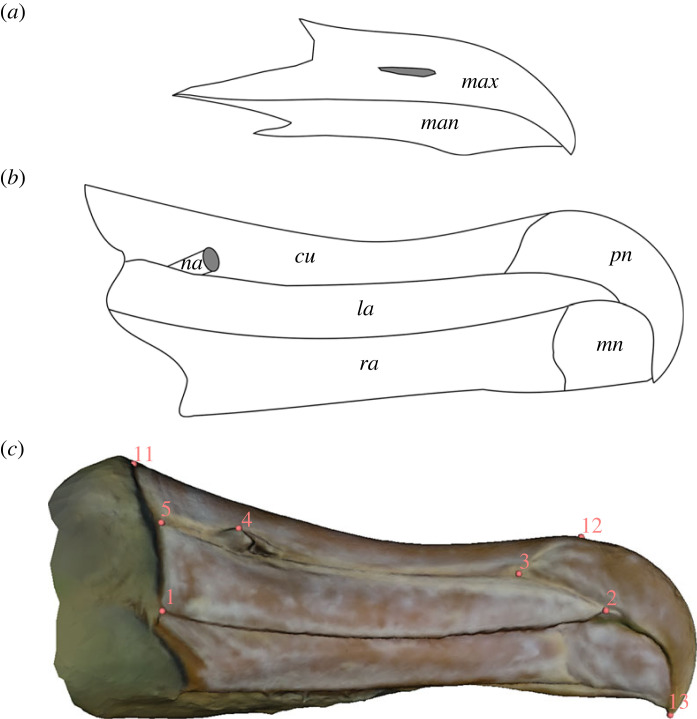
Compound Bill Morphology & Landmark Configuration. (*a*) Bill morphology of European herring gull (*Larus argentatus*) (*b*) Bill morphology of Wandering albatross (*Diomedea exulans*) (*c*) Landmarks 1–5 and 6–10 are paired (right-left) and landmarks 11–13 are found on the midline ( table 1 for descriptions). Specimen B5348 (Campbell Albatross *Thalassarche impavida*). Abbreviations for A & B: max maxilla, man mandible, na naricorn, cu culminicorn, la latericorn, pn premaxillary nail, ra ramicorn, mn mandibular nail.

Albatross (Diomedeidae) are the largest flying birds on Earth. They are pelagic specialists, with several adaptations across their morphology including extreme wingspans and complex bill structures [1–3]. Their wingspans and high wing aspect ratio provide the ability for highly efficient soaring, allowing them to easily travel large distances from their colonies and avoid intense competition with other marine predators, while their charismatic tubenoses are used to filter seawater for drinking [1–3]. These adaptations allow albatross to spend years on the open ocean without making landfall [1–3]. Albatross, along with other members of the seabird order Procellariiformes, have a unique overarching bill structure. Rather than having a single piece of keratin forming the rhamphotheca as in the vast majority of bird species (figure 1*a*), it is constructed out of several plates of keratin to form a compound bill (figure 1*b*) [4–6].

**Table 1.** Landmark descriptions. Landmarks 1–5 and 6–10 are paired (right-left) and landmarks 11–13 are found on the midline. Figure 1*c* for visual.

#	description
1	posterior of latericorn along functional surface (right)
2	anterior tip of latericorn (right)
3	premaxillary nail, latericorn & culminicorn suture point on culminolabial groove (right)
4	naricorn extreme (right)
5	posterior of nasiolabial groove (right)
6	posterior of latericorn along functional surface (left)
7	anterior tip of latericorn (left)
8	premaxillary nail, latericorn & culminicorn suture point on culminolabial groove (left)
9	naricorn extreme (left)
10	posterior of nasiolabial groove (left)
11	posterior of culminicorn (central)
12	culminiolabial groove (central)
13	distal tip of premaxillary nail (central)

**Figure 2.**
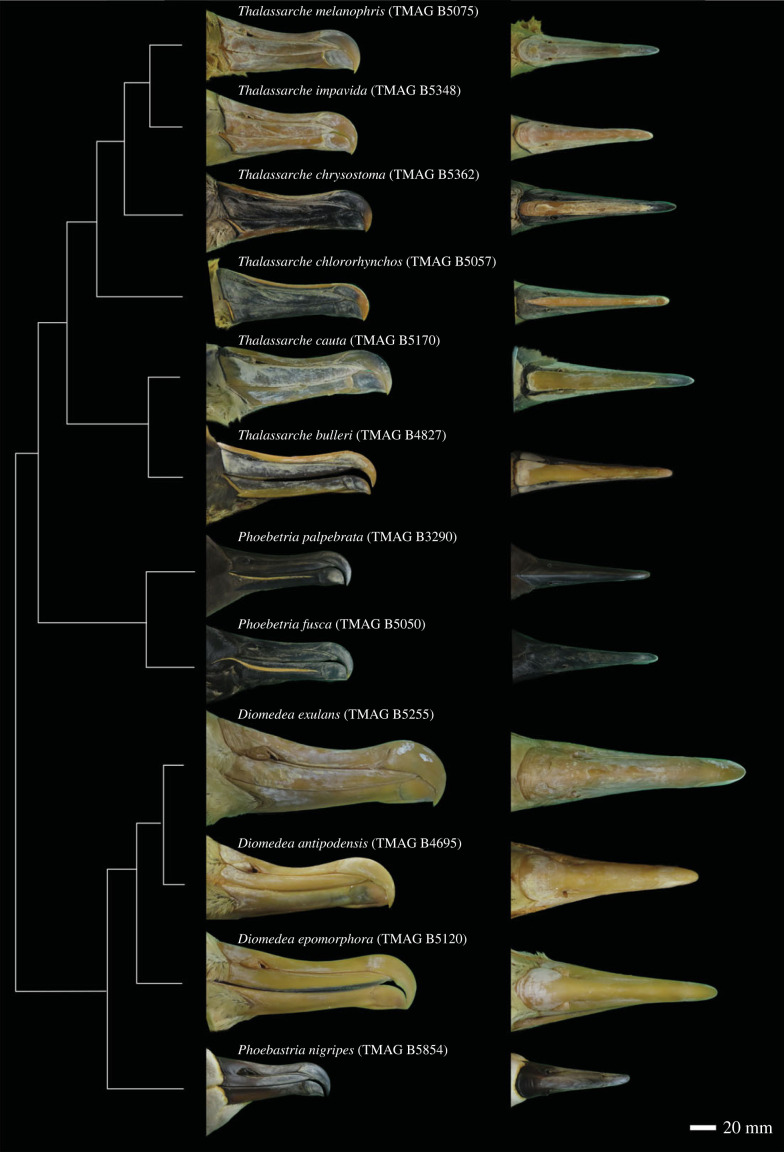
Albatross study species. Cladogram showing the 12 species included in the analyses alongside dorsal and lateral images of representative specimens. Green coloration due to image processing. Original specimen labels for TMAG B4827 & TMAG B5854 listed *Diomedea bulleri* and *Diomedea nigripes* respectively.

Despite their highly specialized pelagic niches, albatross do show interspecific variation across the anatomy, including differences in their bill shape (figure 2) [2,3]. Body size is clearly an important factor in aerodynamics and therefore the evolution of the albatross body plan, but its influence on more specific anatomical structures, such as bill shape, has often been overlooked [6–8]. Allometry describes the relationship between changes in a measurable trait with changes in size and is an inherently intrinsic driver of variation. The presence of a strong allometric signal can be an indicator that the bill structure is experiencing evolutionary constraints limiting variation within species, while a lack of allometry could point towards more extrinsic drivers of shape [9]. We are interested in the relationship of size and bill shape both within species (ontogenetic allometry) and between species (evolutionary allometry) to understand the relative importance of intrinsic factors at different taxonomic levels [10].

The foraging ecology of many albatross species is closely monitored given their conservation risk [3,11]. During the breeding season, they operate as central place foragers but during the non-breeding season, this restriction is lifted and the foraging ranges for albatross species grow to almost oceanwide, with many species' ranges overlapping geographically [12–15]. Albatross diets cover a range of prey including cephalopods, fish, crustaceans, jellyfish and other invertebrates, all found within the upper metres of the ocean surface, with some observations of opportunistic feeding on carrion [16–18]. Variation in avian bill shape has been found to relate to divisions in foraging strategy in many taxa [19–21], therefore, understanding how albatross species are segregating in diet to avoid interspecific competition may provide helpful insight into differences in phenotype, particularly in relation to bill shape. This raises the question of whether albatross bills and their associated keratinous plates are shaped through intrinsic constraints following allometric relationships or driven by extrinsic factors like diet and foraging ecology. Is the variation purely a function of body size differences or do they represent eco-morphological adaptations to minimize interspecific competition?

Species identification and assignment in albatross is a complex challenge, especially for birds caught as fisheries bycatch far from their breeding colonies [22]. Over the last few decades, several studies have sought to clump and split species as advances in integrative taxonomic frameworks have evolved [23–28]. There are currently between 13 and 24 recognized species across four genera, with varying levels of sub-species assignment depending on the bird checklist [3,29–31]. Species identifications of wild albatross can be extremely difficult, with plumage coloration and size being key areas of evidence. In museum collections, this can be further complicated by preservation methods and historic species assignments. Shape and size variation in the bill could therefore be a useful tool for species identification, if species form discrete groups in the trait space.

In this study, we use three-dimensional scans of albatross bills to assess patterns and drivers of shape variation. In particular, we address the following questions: (i) What are the key shape differences between albatross species, and can they be used for species delimitation? (ii) Do extrinsic and intrinsic factors like diet and size drive shape variation? (iii) What influence does phylogeny have in partitioning the morphospace of albatross bills?

## 2. Material and methods

### 2.1. Data collection and photogrammetry of museum specimens

Using specimens from the zoological collection at the Tasmanian Museum and Art Gallery, we sampled 61 individuals from 12 species of albatross covering all 4 genera, predominantly targeting Southern Ocean species (figure 2). These included the Antipodean albatross (*Diomedea antipodensis*, *n* = 1), Southern Royal albatross (*Diomedea epomophora*, *n* = 3), Wandering albatross (*Diomedea exulans*, *n* = 10), Black-footed albatross (*Phoebastria nigripes*, *n* = 1), Sooty albatross (*Phoebetria fusca*, *n* = 4), Light-mantled albatross (*Phoebetria palpebrata*, *n* = 4), Buller's albatross (*Thalassarche bulleri*, *n* = 2), Shy albatross (*Thalassarche cauta*, *n* = 6), Yellow-nosed albatross (*Thalassarche chlororhynchos*, *n* = 8), Grey-headed albatross (*Thalassarche chrysostoma*, *n* = 8), Campbell albatross (*Thalassarche impavida*, *n* = 5) and Black-browed albatross (*Thalassarche melanophris*, *n* = 9). While we have included most Southern Ocean taxa, we recognize that we have a much smaller sample of the North Pacific species, with only one of the four *Phoebastria* species included. The results are therefore focused on Southern Ocean interpretations. For each individual, the upper bill was photographed as a representation of the functional surface of the bill. Each specimen was placed on a turntable within a lightbox, set 1 m away from the camera (Canon SX70HS, resolution: 20.3 MP). A minimum of 62 images (4 concentric rings of 18 photos) were then taken by rotating the specimen 20° and photographing from four different heights (perpendicular to the specimen and 30°, 60° and 75° from the horizontal). Additional photographs were taken of the bill tip when the original 62 did not provide adequate coverage. This allowed the entire bill surface to be imaged with overlap between every photograph (i.e. every location on the bill appears in at least two photos to act as a tie-point in the photogrammetric reconstruction). A scaled three-dimensional model was constructed within Agisoft Metashape, with each textured model being decimated to approximately 50 000 vertices for consistency [32]. Models were then scaled to the nearest millimetre based on scales present in the images. The sample included a range of sizes for each species to reflect the change in size associated with ontogeny. All relevant permissions for handling the specimens were sought from the Tasmanian Museum and Art Gallery and granted.

### 2.2. Geometric morphometric analysis

Each scaled three-dimensional model was imported into Slicer3D [33] and 13 type-I landmarks were applied (figure 1*c*, table 1). All subsequent analysis was conducted within R [34] using the ‘geomorph’ and ‘RRPP’ packages [35–37]. Once all specimens were landmarked, a generalized Procrustes alignment (GPA) was implemented using the ‘gpagen’ function and the symmetric component of shape was retained for further analyses (symmetric shape = 92% total variation; fluctuating asymmetry = 8% total variation). Principal components analysis (PCA) was used to construct a bill morphospace for all specimens using the ‘gm.prcomp’ function and all axes were retained for analysis. Three-dimensional warped meshes and wireframes were constructed to visualize the shape variation along PC axes using the *plotRefToTarget* function (electronic supplementary material, B).

### 2.3. Testing association between bill shape, ecology and size

To test for relationships between bill shape and ecology and size, we used the ‘procD.lm’ function to perform Procrustes ANOVAs and MANOVAs. Diet data was collated from the EltonTraits database [38] and includes a breakdown of proportion of invertebrates, proportion of fish and proportion of scavenging in the diet for each species. These proportions are then collated into one of three categories: Invertivore (66%+ invertebrates) or Vertebrate-Fish-Scavenging (66%+ fish & scavenging) or Omnivore (less than 66% in all categories). Only the Black-footed albatross is observed using scavenging in the data, with the remaining species consuming a mix of invertebrates or fish in inverse proportions. In the subsequent analyses, we therefore only use invertebrate proportion to reduce redundancy. Centroid size was extracted from the landmark data and was log-transformed for use in further analyses. We produced a size-shape PCA using the ‘plotAllometry’ function with the ‘*size.*shape’ method which combines the landmark data and centroid size data into a single matrix and performs a PCA, thereby reintroducing the size variable that was removed via the GPA. To test patterns in evolutionary and ontogenetic allometry, we fit three different linear models: 1) a Simple Allometric Model (shape ∼ Centroid Size) where all taxa follow the same allometry vector, 2) a Common Allometric Model (shape∼Centroid Size+species)  where the vectors of shape change are parallel and the mean predictions are different (i.e. there is no interaction term with each species sharing the same gradient but having different intercepts), and 3) a Unique Allometric Model (shape∼Centroid Size+species+Centroid Size ∗ species)  where the gradients and intercepts for each species are different (i.e. inclusion of an interaction parameter). We used the homogeneity of slopes (HOS) test, using the ‘*anova.lm.rrpp’* function, to compare the three different models with a significant result indicating that the allometric relationship is not the same for at least one species, either in terms of the species mean or the gradient of the regression line. In this case, we tested models sequentially in order of increasing complexity to test the inclusion of species means, excluding and then including the interaction term (i.e. Simple Vs Common, then Common Vs Unique). The associated regressions, visualized in figure 6, show if the individuals sat on the regression lines with no residuals. In this case, the y-axis is the first axis of a PCA on the expected fitted values of each individual (i.e. the model was a perfect fit). We also ran the simple allometry model for subsets of the data including for each species and for each genus.

### 2.4. Species averaging and phylogenetic analyses

To understand the influence of phylogeny, we averaged the landmark configurations of all specimens per species (excluding those that were identified as chicks) and then produced a new PCA onto which we mapped the phylogeny. The phylogenetic relationships were taken from the maximum clade credibility (MCC) tree of the Hackett backbone [39] where the total bird tree was pruned to only the 12 species included in the analyses. To understand the phylogenetic signal, we used the ‘*physignal*’ function to calculate Blomberg's K [40,41] for both species shape and size. *K* = 1 suggests that the signal strength is equal to that expected under Brownian Motion. Larger values mean that the taxa are more alike than expected under Brownian Motion with the opposite being true for *K* < 1. For shape, the value of *K* was calculated for all axes of the phylogenetically aligned components analysis and also for each increasing dimension (*K* by *p*: i.e. 1, 1 : 2, 1 : 3) to understand how the addition of each dimension affects the signal.

## 3. Results

### 3.1. Shape variation in albatross bills

**Figure 3.**
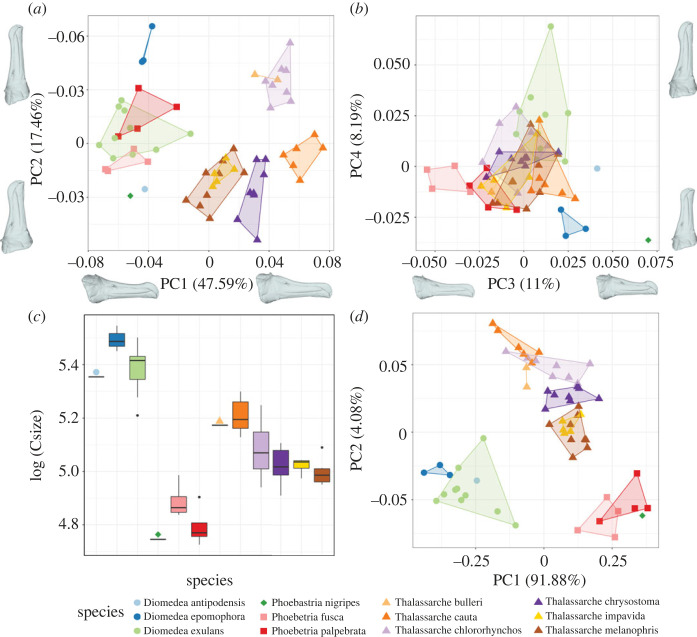
Shape and size. (*a*) Morphospace of first two principal components with convex hulls around species. (*b*) Morphospace of the third and fourth principal components with convex hulls around species. (*c*) Boxplot showing ranges of bill centroid size (key symbols used to identify bars where *n* = 1 or *n* = 2). (*d*) First two axes of the Size-Shape morphospace based on the 13 landmarks and centroid size data.

**Figure 4.**
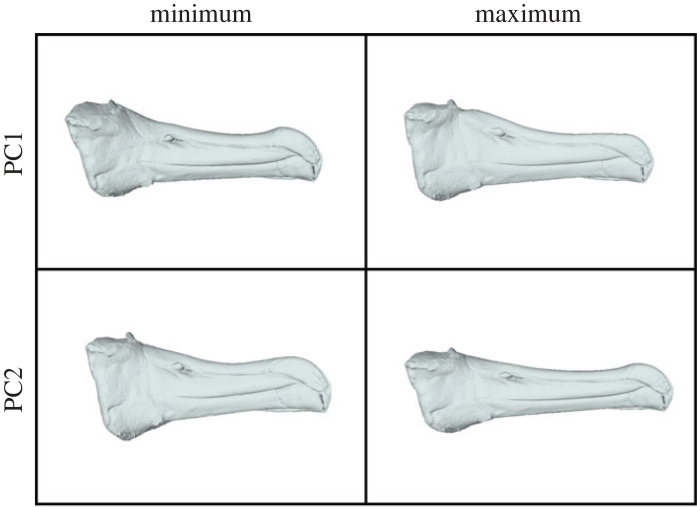
Relative Warps. Specimen B5348 (Campbell Albatross *Thalassarche impavida*) warped to the average shape and then to the extremes of Principal Component 1 & 2.

Variation across the albatross bill is split across several key axes of variation. The first principal component (47.59%) separates the *Thalassarche* genus from the *Diomedea, Phoebetria* and *Phoebastria* genera, with individuals from wandering & shy albatross representing the extreme positions. The axis is dominated by variation at the anterior and posterior of the bill, in particular the relative contribution of the latericorn and culminicorn to the overall caudal thickness. Individuals with more negative PC1 scores have thinner latericorns and thicker culminicorns caudally and more upright premaxillary nail sutures versus individuals with positive scores having much thicker latericorns, thinner culminicorns and elongate premaxillary nail sutures. The variation caudally also has an impact on the relative position of the nares, being more dorsal and posterior at negative scores and more centred at positive scores. The second principal component (17.46%) describes the relative elongation of both the latericorn and culminicorn and more broadly a change in the aspect ratio. The plates are proportionally longer and thinner for individuals with positive PC2 scores and shorter and thicker for those with negative scores (figures 3*a* and 4, electronic supplementary material, B). In this case, there are no obvious taxonomic splits, instead the extremes are occupied by a range of species. PC3 & PC4 account for smaller amounts of the overall variation (11.00% and 8.19% respectively; figure 3*b*, electronic supplementary material, B). PC3 relates to the relative curvature of various sutures while PC4 is dominated by the relative proportion of the premaxillary nail. This fourth axis is dominated by a single individual outlier at the positive extreme which represents a *Diomedea exulans* chick. Most taxa are well differentiated in the PC1-PC2 morphospace (figure 3*a*), but that separation is lost in the PC3-PC4 morphospace (figure 3*b*). Bill size falls into three clusters, split at the genera level, with *Diomedea* being the largest*, Phoebetria* & *Phoebastria* being the smallest and *Thalassarche* occupying an intermediate range (figure 3*c*).

When both size and shape are accounted for, all genera are clearly partitioned in morphospace (figure 3*d*). Procrustes MANOVAs of shape and size against species are both highly significant, with species means accounting for 90% of the variation in size and 79% of the variation in shape (*p* = 0.001 for both tests, figure 3*a–c*). Electronic supplementary material, C contains the pairwise comparisons between species for both shape and size.

### 3.2. Evolution of bill shape in relation to ecology

**Figure 5.**
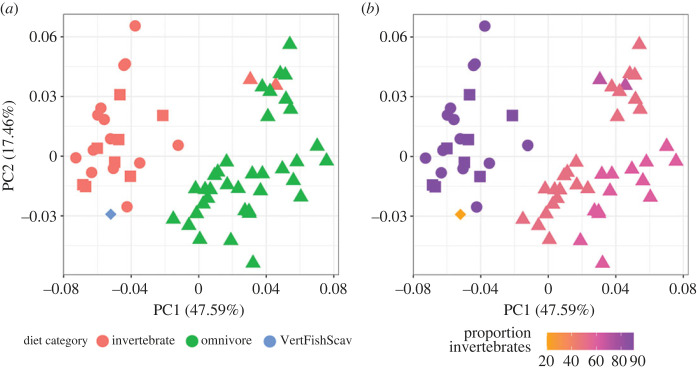
Diet. Both plots present first two PCA axes matching figure 4A with corresponding shapes mapping to genera (Circle – *Diomedea*, Diamond – *Phoebastria*, Square – *Phoebetria*, Triangle – *Thalassarche*). (*a*) Morphospace showing three discrete dietary categories: Invertivore, Omnivore, Fish and Scavenging (*b*) proportion of invertebrates in diet as given by the EltonTraits database.

Each albatross species was assigned to one of three broad dietary categories: Omnivore, Invertivore & Vertebrate Fish Scavenger. Figure 5*a* shows these categorizations mapped onto the first two axes of the Principal Component space. Omnivores occupy positive PC1 space with both the more specialist diets (invertivores and fish-scavengers) occupying negative PC1 space. Both Omnivores and Invertivores are fully spread across PC2 with the fish eating *Phoebastria* in the lower left quadrant.

Overall, bill size and shape differ significantly among the three diet classes. Procrustes ANOVAs for shape and regular ANOVAs for size were both significant (shape: *F* = 18.092, *p* = 0.001, *R*^2^ = 0.38418; size: *F* = 5.6404, *p* = 0.006, *R*^2^ = 0.16283). Pairwise comparisons of bill shape were all significant (Invertebrate:Omnivore *p* = 0.001, Invertebrate:VertFishScav *p* = 0.015, Omnivore:VertFishScav *p* = 0.001); as were most pairwise comparisons of bill size (Invertebrate:Omnivore *p* = 0.014, Invertebrate:VertFishScav *p* = 0.010), with the exception of Omnivores and Fish Scavengers which did not differ significantly in size (*p* = 0.172). When the proportions of diet are used, we find that bill shape and invertebrate diet proportion are significantly correlated based on an ANOVA test (*F* = 20.235, *p* = 0.001, *R*^2^ = 0.2553, figure 5*b*), but has lower *R*^2^ values than the coarse diet categorization.

### 3.3. Allometric patterns within and between species

**Figure 6.**
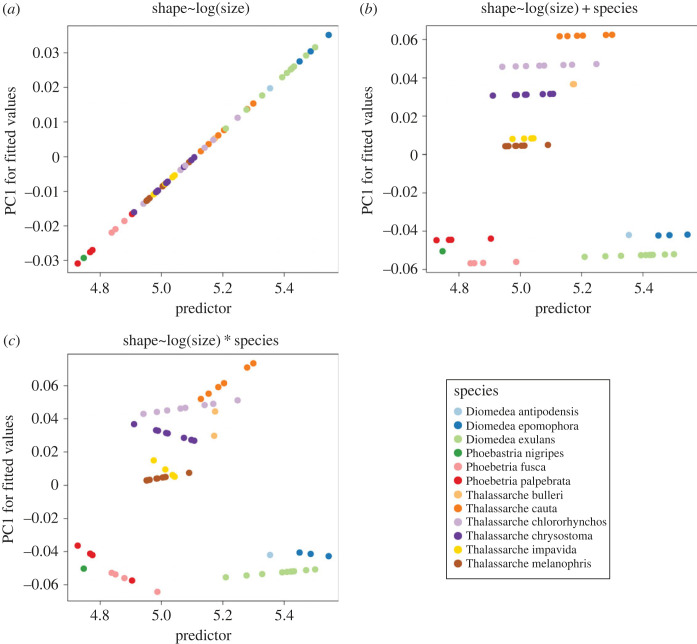
Comparison of three proposed allometric models predicting albatross bill shape. To test which allometric model best describes variation observed within albatross, three potential models (*a*–*c*). X-axis plots centroid size and y-axis plots Principal Component 1 of the fitted values of predicted shape (i.e. excluding the residuals) from the proposed regression. These panels are the equivalent of an R^2^ equal to 1. (*a*) Simple Allometric Model (shape∼Centroid Size) where all taxa follow the same allometry vector (*b*) Common Allometric Model (shape∼Centroid Size+species) where the gradients of shape change are the same but the means are different (i.e. each species shares the same gradient but have different intercepts) (*c*) Unique Allometric Model (shape∼Centroid Size ∗ species) where the gradients and intercepts for each species are different (i.e. inclusion of an interaction parameter) (*d*) Legend for plots (*a*–*c*).

Allometry in geometric morphometrics uses linear models to predict shapes and construct shape change vectors using size and other covariates. In the simplest case, the predicted shape of an individual bill is dependent only on its size (Simple Allometric Model) while more complex models include predictions based on species assignment (Common Allometric Model) and allow interactions between covariates to produce different shape change vectors (Unique Allometric Model). We compared these three models, incorporating species means and associated interaction parameters alongside size, using a Homogeneity of Slopes test (described in the Methods and Materials and visualized in figure 6). Positive and negative gradients in this context translate to having a certain vector of shape change associated with size and the steeper the gradient, the greater the shape variation with a unit change in size. We found that the Common Allometric Model (figure 6*b*) produced a significant result when compared to the Simple Allometric Model (figure 6*a*), yet there was not significant support to accept the Unique model (figure 6*c*) (Simple versus Common; *p* = 0.001 & Common versus Unique; *p* = 0.266) (electronic supplementary material, D). This result translates to each species having a different predicted mean shape at a given centroid size, but the vector of shape change is common across all species with respect to size. The Common model accounts for approximately 80% of the variation with differences between species mean shapes explaining 10 times more than the size component (species: *F* = 15.432, *p* = 0.001, *R*^2^ = 0.726; size: *F* = 15.926, *p* = 0.001, *R*^2^ = 0.068) (electronic supplementary material, D). The gradients of allometric shape change in the Common model are also very shallow indicating very small shape changes across the size ranges occupied by the albatross bills, approaching isometric growth (i.e. no shape change vector associated with size). Each species was subset and tested for significant allometry and only two species returned significant results (*Thalassarche impavida* - *R*^2^ = 0.527, *p* = 0.017, & *Thalassarche chrysostoma* - *R*^2^ = 0.328*, p* = 0.048). The test was repeated at the genera level and both *Phoebetria* and *Thalassarche* have significant results (*Phoebetria* - *R*^2^ = 0.373*, p* = 0.013, and *Thalassarche - R*^2^ = 0.190*, p* = 0.001). In all of the significant results, the goodness of fit values were higher than in the Common model fit (*R*^2^ = 0.068).

### 3.4. Phylogenetic signal in species average shape

**Figure 7.**
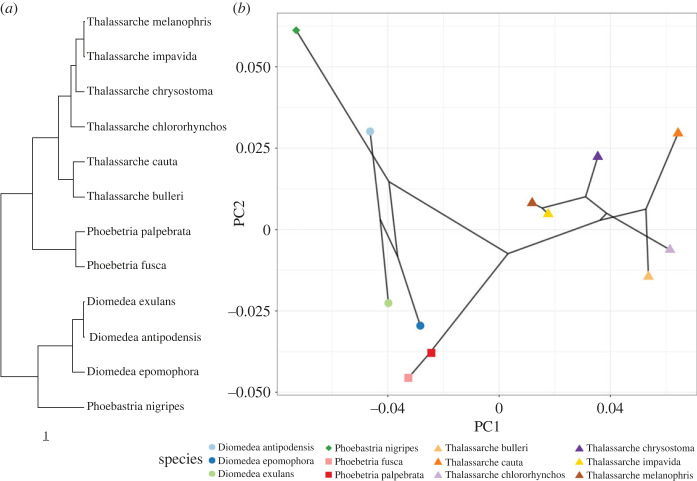
Phylogeny and Phylomorphospace. (*a*) Scaled albatross phylogeny used for analysis (*b*) Phylomorphospace based on PCA analyses of species average bill shape. Colours and shapes match figure 3.

By averaging the bill shape for each species, we were able to understand the relative importance of phylogeny (figure 7*a*) in driving the shape and size variation. Fitting Blomberg's *K* to both shape and size data returned statistically significant results for both *K* = 0.29 and 1.10, *p* = 0.001 and *p* = 0.001, respectively. This implies that there is greater divergence in bill shape phylogenetically (compared to expectations under Brownian Motion), whereas bill size varies phylogenetically approximately as expected under Brownian Motion, i.e. more closely related taxa have relatively similarly sized bills, but greater than expected differences in shape. Another interpretation of the signal in shape is that the variance is found more within clades than between them. The *K* by *p* sequence shows that the first axis of shape has a remarkably high *K* value for that axis alone (*K* = 1.9) but that is greatly reduced by the addition of subsequent axes (table 2).

**Table 2.** *K* by *p* values. For each additional dimension, *K* is recalculated (*K* by *p*: i.e. 1, 1 : 2, 1 : 3) until all dimension are included which matches the overall *K* value calculation.

# of axes	*K* value
1	1.907207
2	0.601233
3	0.474638
4	0.427955
5	0.389136
6	0.354779
7	0.344101
8	0.339541
9	0.338771
10	0.297675
11	0.29136
12	0.29136

## 4. Discussion

### 4.1. Albatross bill size and shape variation

Here we show that albatross species are phenotypically divergent in both the shape and size of their bills. The majority of species and genera are clearly delineated by the first two principal components of the bill shape morphospace (figure 3*a*), which together explain over 65% of bill shape variation. The bills of *Thalassarche impavida* (Campbell's albatross) and *Thalassarche melanophris* (Black-browed albatross) have complete overlap in both the shape and size-shape morphospaces (figure 3*a,c*). *Thalassarche impavida* was historically considered a subspecies of *Thalassarche melanophris*, until it was elevated to a full species following genetic analyses [25]. Our finding reinforces the phenotypic similarity of the two taxa and is unsurprising given their shallow phylogenetic divergence [25]. Both the *Phoebetria species* (Sooty and Light-mantled albatross) overlap with *Diomedea exulans* (Wandering albatross) in shape morphospace. However, the inclusion of size into the morphospace (figure 3*d*) completely separates the *Phoebetria* species from the *Diomedea* species, suggesting convergence in bill shape but divergence in size to occupy different foraging niches. This is achieved by eating proportionally sized prey, despite their dietary and range overlap in the South Atlantic and South Indian sections of the Southern Ocean between 40° and 60° South (electronic supplementary material, E) [3,42–44]. This is reinforced by the results of the pairwise comparisons of shape and size between species. *Phoebetria palpebrata* is not statistically different in shape from any of the *Diomedea* species (*p* > 0.11) but is significantly different in size (*p* < 0.01) (figure 3*c*, electronic supplementary material, C). The use of the bill for species identification seems well founded, with 50 of the 66 pairwise comparisons recovering a statistically significant difference in either shape or size or both. Of the comparisons that were non-significant, 10 included species that only had one or two individuals in the dataset. Where sample sizes were high, species were readily discernible by one or both features.

### 4.2. Diet as an explanatory factor for bill shape

We found that both extrinsic (diet) and intrinsic (size) factors are found to play a role in driving upper bill shape variation. Species, diet, and size are all statistically significant predictors of an individual's bill shape, with species the strongest predictor. Diet was also found to be a significant predictor of bill shape, particularly in the case of the differences between omnivores and invertivores.

Specialist invertivores and fish-scavengers dominate the negative PC1 region of the morphospace, with the generalist omnivores occupying the positive PC1 region (figure 5*a*). When we break down the ‘generalist omnivore’ category to consider the specific dietary proportions a slightly different pattern emerges. Taxa that consume a 50 : 50 split of invertebrates and fish sit in a valley between increasing invertebrate proportion; *Diomedea* and *Phoebetria* both consume high (90%) proportions of invertebrates at the negative PC1 extreme while *T. chrysostoma* and *T. cauta* show a much smaller increase (60%) in their invertebrate intake and occupy the lower right quadrant of the PC1-PC2 morphospace. One oddity is the position of the Black-footed albatross (*Phoebastria nigripes*), here represented by a single individual. The Black-footed albatross is a North Pacific species and its dietary contents are known from a small number of studies, all indicating that a large proportion of its diet consists of flying fish eggs [45,46]. Therefore, while its diet consists of high proportions of fish (figure 5*a*,*b*), the actual material being ingested is very different from the whole fish consumed by the *Thalassarche*. The other prominent exception is the position of the invertivore Buller's albatross relative to the other invertivores. The morphospace shows a clear phylogenetic split between the *Thalassarche* genus and the other three included in the analyses. Therefore, the strength of phylogenetic conservatism may in this case be stronger than the extrinsic ecological pull to convergent morphologies, which has been documented in other specific bird groups but appears to be rarer at larger scales [19,20,47,48].

### 4.3. Allometry

The influence of size on bill shape seems dependent on the taxonomic level, with both evolutionary and ontogenetic allometry signals being weak or sparse throughout the group. The results reiterate that of the intrinsic factors analysed here, species assignment is far more predictive than size. At the family level, based on the Homogeneity of Slopes testing, the Common Allometric Model best predicts bill shape, which implies that the shape change vector is shared across all species. This model however found that the predictive power of size was 10 times smaller than species assignment so despite both being significant predictors, the size signal is relatively weak. More interesting are the results from lower taxonomic ranks which point towards differing allometric patterns across the phylogeny. Only two species, *Thalassarche impavida* and *Thalassarche chrysostoma*, returned a significant allometric signal when each species was examined individually but when considering the genera level, two of the three Southern Ocean clades (*Thalassarche* and *Phoebetria*) had significant signals. This lack of signal in *Diomedea* alongside the shallow gradients of shape change found in the linear models points towards a predominantly isometric model of growth ontogenetically in the Great albatross. Moreover, the predictive power of size in the allometric models decreases as the overall centroid size of the genera increases (Phoebetria *R*^2^ = 0.373, Thalassarche *R*^2^ = 0.190, Diomedea *R*^2^ = 0.141 (non-significant)), suggesting that allometric constraint is stronger at smaller sizes.

### 4.4. Phenotypic differences mirror phylogenetic relationships

The strong phylogenetic patterns in figure 7*b* and the *K* by *p* sequence point to deeper divergences in the past in terms of bill shape in albatross. The large first *K* value in the sequence reflects the split at the root, seen in figure 7*b*, delineating genera on PC1. It suggests that the differences between genera are far more pronounced than those seen between species. Indeed, the Phylomorphospace (figure 7*b*) shows how sister taxon are diverging in shape space, most likely through competitive displacement given range overlaps but other more distant related taxa are converging on common forms. This is most pronounced in between the *Diomedea* and *Phoebetria* species.

## 5. Conclusion

Here we have constructed one of the first three-dimensional studies focused on the albatross compound bill. Despite being the largest seabirds, size is a relatively unimportant factor in the evolution of the albatross bill. Species means and coarse diet categorisation explain far more of the variation, as indicated by the ANOVA and Homogeneity of Slopes testing. We do find however that albatross species are indeed partitioning through differing shape and size to utilize similar resources, in this case invertivore specialists, while avoiding direct competition. The results show that both intrinsic and extrinsic factors should be considered when understanding morphological evolution. Three-dimensional data collection on at risk birds will be vital to understand their morphological adaptations and particularly in albatross where the notion of a species is more complex.

## Data Availability

All data are available in the electronic supplementary material, A [49].
